# 2-(4-Bromo­phen­yl)-1-pentyl-4,5-diphenyl-1*H*-imidazole

**DOI:** 10.1107/S1600536813021983

**Published:** 2013-08-14

**Authors:** Shaaban K. Mohamed, Mehmet Akkurt, Kuldip Singh, Adel A. Marzouk, Mustafa R. Albayati

**Affiliations:** a Chemistry and Environmental Division, Manchester Metropolitan University, Manchester M1 5GD, England; b Chemistry Department, Faculty of Science, Minia University, 61519 El-Minia, Egypt; c Department of Physics, Faculty of Sciences, Erciyes University, 38039 Kayseri, Turkey; d Department of Chemistry, University of Leicester, Leicester, England; e Pharmaceutical Chemistry Department, Faculty of Pharmacy, Al Azhar University, Egypt; f Kirkuk University, College of Science, Department of Chemistry, Kirkuk, Iraq

## Abstract

The title compound, C_26_H_25_BrN_2_, is isomorphous with the chloro derivative [2-(4-chloro­phen­yl)-1-pentyl-4,5-diphenyl-1*H*-imidazole; Mohamed *et al.* (2013 ▶). Acta Cryst. E**69**, o846–o847]. The two phenyl rings and the 4-bromo­phenyl ring are oriented at dihedral angles of 30.1 (2), 64.3 (3) and 42.0 (2)°, respectively, with respect to the imidazole ring. In the crystal, mol­ecules stack in columns along the *b*-axis direction, however, there are no significant inter­molecular inter­actions present.

## Related literature

For biological and synthetic applications of imidazole deriv­atives, see: Maier *et al.* (1989*a* ▶,*b* ▶); Welton (1999 ▶); Hermann & Kocher (1997 ▶). For related structures, see: Akkurt *et al.* (2013 ▶); Mohamed *et al.* (2013 ▶); Simpson *et al.* (2013 ▶).
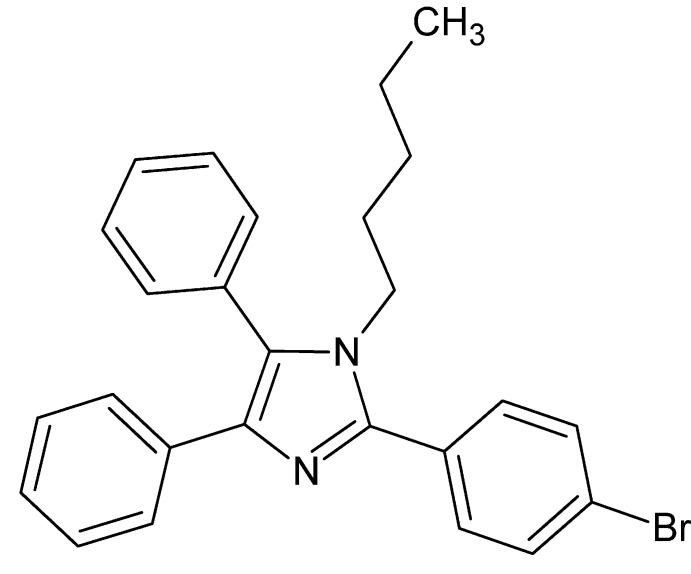


## Experimental

### 

#### Crystal data

C_26_H_25_BrN_2_
*M* *_r_* = 445.39
Monoclinic, 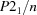

*a* = 10.665 (5) Å
*b* = 9.619 (5) Å
*c* = 21.541 (10) Å
β = 91.092 (9)°
*V* = 2209.4 (19) Å^3^
*Z* = 4
Mo *K*α radiation
μ = 1.88 mm^−1^
*T* = 150 K
0.36 × 0.16 × 0.03 mm

#### Data collection

Bruker APEXII CCD area-detector diffractometer
Absorption correction: multi-scan (*SADABS*; Bruker, 2011 ▶) *T*_min_ = 0.600, *T*_max_ = 0.969
16966 measured reflections
4329 independent reflections
2328 reflections with *I* > 2σ(*I*)
*R*_int_ = 0.187

#### Refinement

*R*[*F* ^2^ > 2σ(*F* ^2^)] = 0.066
*wR*(*F* ^2^) = 0.116
*S* = 0.88
4329 reflections
263 parameters
H-atom parameters constrained
Δρ_max_ = 0.61 e Å^−3^
Δρ_min_ = −1.05 e Å^−3^

### 

Data collection: *APEX2* (Bruker, 2011 ▶); cell refinement: *SAINT* (Bruker, 2011 ▶); data reduction: *SAINT*; program(s) used to solve structure: *SHELXS97* (Sheldrick, 2008 ▶); program(s) used to refine structure: *SHELXL97* (Sheldrick, 2008 ▶); molecular graphics: *ORTEP-3 for Windows* (Farrugia, 2012 ▶); software used to prepare material for publication: *WinGX* (Farrugia, 2012 ▶) and *PLATON* (Spek, 2009 ▶).

## Supplementary Material

Crystal structure: contains datablock(s) global, I. DOI: 10.1107/S1600536813021983/su2633sup1.cif

Structure factors: contains datablock(s) I. DOI: 10.1107/S1600536813021983/su2633Isup2.hkl

Supplementary material file. DOI: 10.1107/S1600536813021983/su2633Isup3.cml

Additional supplementary materials:  crystallographic information; 3D view; checkCIF report

## Figures and Tables

**Fig. 1.**
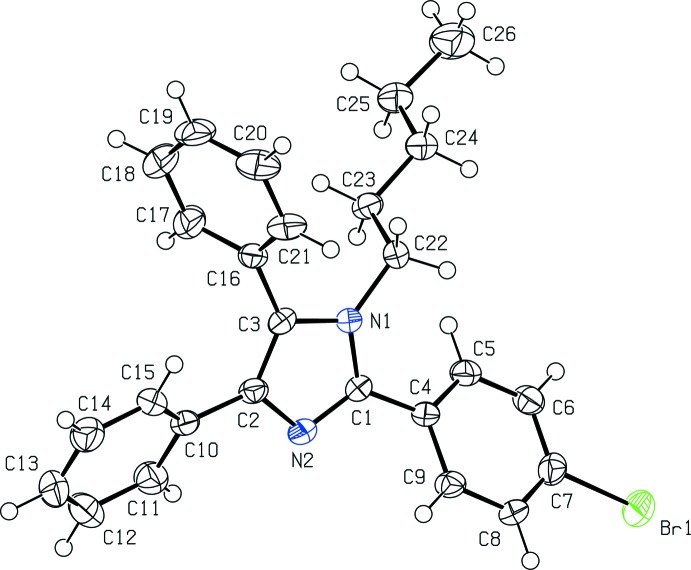
The molecular structure of the title molecule, with atom labelling. Displacement ellipsoids are drawn at the 50% probability level.

**Fig. 2.**
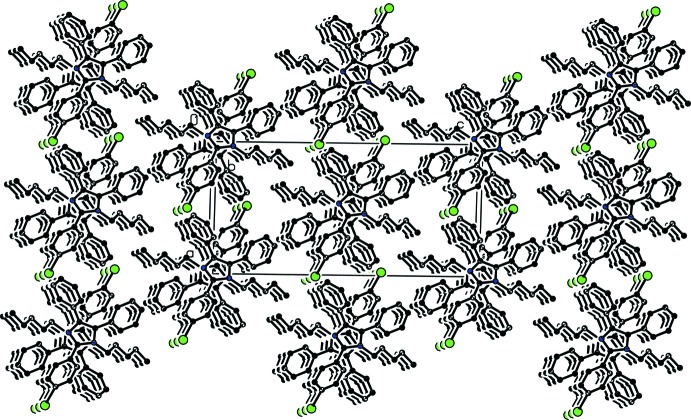
The crystal packing of the title compound, viewed along the b axis.

## supplementary crystallographic information

### 1. Comment

Many substituted imidazoles exhibit diverse pharmaceutical properties and are known inhibitors of fungicides and herbicides, plant growth regulators and therapeutic agents (Maier *et al.*, 1989**a*,b*). Moreover, they are of interest for environmental and green chemistry applications, and have been prepared and used as a large class of ionic liquids and Lewis base catalysts (Welton, 1999; Hermann & Kocher, 1997). As part of our ongoing study of the synthesis (Akkurt *et al.*, 2013; Mohamed *et al.*, 2013; Simpson *et al.*, 2013) and biological applications of tetrasubstituted imidazoles, we herein report on the synthesize and crystal structure of the title compound.

The title compound, Fig. 1, and the chloro derivative, 2-(4-Chlorophenyl)-1-pentyl-4,5-diphenyl-1*H*-imidazole, whose crystal structure has been reported by (Mohamed *et al.*, 2013), are isomorphous. In the title compound the phenyl rings (C10–C15 and C16–C21) and the 4-bromophenyl ring (C4–C9) make dihedral angles of 30.1 (2), 64.3 (3) and 42.0 (2)°, respectively, with the imidazole ring (N1/N2/C1–C3). The values of the bond lengths and bond angles fall within the normal range and are comparable with those reported for similar structures (Akkurt *et al.*, 2013; Mohamed *et al.*, 2013; Simpson *et al.*, 2013).

In the crystal, the molecules stack in columns along the b axis direction however, there are no significant intermolecular interactions present (Fig. 2).

### 2. Experimental

The title compound was synthesized following our previously reported procedure (Mohamed *et al.*, 2013). Colourless plates of the title compound (*M*.p. 396–398 K) were collected with 84% yield. Crystals of sufficient quality for the X-ray diffraction study were obtained by slow evaporation of an ethanol solution of the title compound.

### 3. Refinement

All H atoms were placed in geometrically idealized positions with C—H = 0.95 - 0.99 Å and refined using a riding model with *U*_iso_(H) = 1.5*U*_eq_(C-methyl) and = 1.2*U*_eq_(C) for other H atoms. The R_int_ value is rather high probably due to the fact that the crystal diffracted weakly beyond 22° in θ.

### Crystal data

**Table:**

C_26_H_25_BrN_2_	*F*(000) = 920
*M**_r_* = 445.39	*D*_x_ = 1.339 Mg m^−^^3^
Monoclinic, *P*2_1_/*n*	Mo *K*α radiation, λ = 0.71073 Å
Hall symbol: -P 2yn	Cell parameters from 638 reflections
*a* = 10.665 (5) Å	θ = 2.3–23.5°
*b* = 9.619 (5) Å	µ = 1.88 mm^−^^1^
*c* = 21.541 (10) Å	*T* = 150 K
β = 91.092 (9)°	Plate, colourless
*V* = 2209.4 (19) Å^3^	0.36 × 0.16 × 0.03 mm
*Z* = 4	

### Data collection

**Table:**

Bruker APEXII CCD area-detector diffractometer	4329 independent reflections
Radiation source: fine-focus sealed tube	2328 reflections with *I* > 2σ(*I*)
Graphite monochromator	*R*_int_ = 0.187
φ and ο scans	θ_max_ = 26.0°, θ_min_ = 1.9°
Absorption correction: multi-scan (*SADABS*; Bruker, 2011)	*h* = −13→13
*T*_min_ = 0.600, *T*_max_ = 0.969	*k* = −11→11
16966 measured reflections	*l* = −25→26

### Refinement

**Table:**

Refinement on *F*^2^	Primary atom site location: structure-invariant direct methods
Least-squares matrix: full	Secondary atom site location: difference Fourier map
*R*[*F*^2^ > 2σ(*F*^2^)] = 0.066	Hydrogen site location: inferred from neighbouring sites
*wR*(*F*^2^) = 0.116	H-atom parameters constrained
*S* = 0.88	*w* = 1/[σ^2^(*F*_o_^2^) + (0.0277*P*)^2^] where *P* = (*F*_o_^2^ + 2*F*_c_^2^)/3
4329 reflections	(Δ/σ)_max_ = 0.001
263 parameters	Δρ_max_ = 0.61 e Å^−^^3^
0 restraints	Δρ_min_ = −1.05 e Å^−^^3^

### Special details

**Table:**

Geometry. Bond distances, angles *etc*. have been calculated using the rounded fractional coordinates. All su's are estimated from the variances of the (full) variance-covariance matrix. The cell e.s.d.'s are taken into account in the estimation of distances, angles and torsion angles
Refinement. Refinement on *F*^2^ for ALL reflections except those flagged by the user for potential systematic errors. Weighted *R*-factors *wR* and all goodnesses of fit *S* are based on *F*^2^, conventional *R*-factors *R* are based on *F*, with *F* set to zero for negative *F*^2^. The observed criterion of *F*^2^ > σ(*F*^2^) is used only for calculating -*R*-factor-obs *etc*. and is not relevant to the choice of reflections for refinement. *R*-factors based on *F*^2^ are statistically about twice as large as those based on *F*, and *R*-factors based on ALL data will be even larger.

### Fractional atomic coordinates and isotropic or equivalent isotropic displacement parameters (Å^2^)

**Table:**

	*x*	*y*	*z*	*U*_iso_*/*U*_eq_	
Br1	0.48280 (5)	1.06999 (7)	1.12186 (2)	0.0518 (2)	
N1	0.9579 (3)	0.7532 (4)	0.95097 (14)	0.0260 (14)	
N2	1.0328 (3)	0.7435 (4)	1.04691 (14)	0.0291 (14)	
C1	0.9420 (4)	0.7928 (5)	1.01128 (18)	0.0266 (14)	
C2	1.1118 (4)	0.6715 (5)	1.00900 (18)	0.0266 (14)	
C3	1.0659 (4)	0.6773 (5)	0.94893 (18)	0.0289 (14)	
C4	0.8327 (4)	0.8632 (5)	1.03554 (18)	0.0287 (14)	
C5	0.7751 (4)	0.9785 (5)	1.00753 (18)	0.0330 (18)	
C6	0.6714 (4)	1.0384 (5)	1.03236 (18)	0.0331 (18)	
C7	0.6244 (4)	0.9856 (5)	1.08588 (19)	0.0340 (18)	
C8	0.6802 (4)	0.8754 (6)	1.11597 (19)	0.0356 (18)	
C9	0.7843 (4)	0.8143 (5)	1.09045 (18)	0.0290 (16)	
C10	1.2222 (4)	0.6036 (5)	1.03414 (18)	0.0286 (16)	
C11	1.2824 (4)	0.6578 (5)	1.0867 (2)	0.0403 (19)	
C12	1.3866 (5)	0.5930 (7)	1.1121 (2)	0.054 (2)	
C13	1.4331 (5)	0.4747 (6)	1.0864 (2)	0.053 (2)	
C14	1.3738 (4)	0.4185 (6)	1.0344 (2)	0.0462 (19)	
C15	1.2697 (4)	0.4841 (5)	1.00872 (19)	0.0344 (18)	
C16	1.1136 (4)	0.6206 (5)	0.89021 (18)	0.0287 (16)	
C17	1.2266 (4)	0.6687 (6)	0.8681 (2)	0.0426 (19)	
C18	1.2736 (5)	0.6162 (6)	0.8134 (2)	0.053 (2)	
C19	1.2084 (5)	0.5199 (6)	0.7801 (2)	0.048 (2)	
C20	1.0959 (5)	0.4708 (6)	0.80118 (19)	0.047 (2)	
C21	1.0490 (5)	0.5224 (5)	0.85652 (18)	0.0366 (16)	
C22	0.8836 (4)	0.7999 (5)	0.89621 (18)	0.0304 (16)	
C23	0.9433 (4)	0.9208 (5)	0.86564 (18)	0.0313 (14)	
C24	0.8586 (4)	0.9869 (5)	0.81709 (19)	0.0360 (18)	
C25	0.9148 (4)	1.1120 (6)	0.7870 (2)	0.048 (2)	
C26	0.8257 (5)	1.1835 (6)	0.7416 (2)	0.064 (2)	
H5	0.80860	1.01570	0.97050	0.0400*	
H6	0.63250	1.11590	1.01260	0.0400*	
H8	0.64750	0.84160	1.15380	0.0430*	
H9	0.82320	0.73770	1.11090	0.0350*	
H11	1.25130	0.74020	1.10520	0.0480*	
H12	1.42660	0.63130	1.14800	0.0640*	
H13	1.50530	0.43110	1.10410	0.0640*	
H14	1.40460	0.33530	1.01640	0.0560*	
H15	1.23010	0.44580	0.97270	0.0410*	
H17	1.27200	0.73790	0.89040	0.0510*	
H18	1.35230	0.64800	0.79910	0.0630*	
H19	1.24060	0.48620	0.74210	0.0580*	
H20	1.05050	0.40230	0.77820	0.0560*	
H21	0.97110	0.48900	0.87110	0.0440*	
H22A	0.79830	0.82590	0.90930	0.0360*	
H22B	0.87570	0.72240	0.86610	0.0360*	
H23A	1.02160	0.88990	0.84590	0.0380*	
H23B	0.96580	0.99100	0.89750	0.0380*	
H24A	0.83830	0.91710	0.78460	0.0430*	
H24B	0.77900	1.01430	0.83670	0.0430*	
H25A	0.99110	1.08340	0.76480	0.0570*	
H25B	0.94060	1.17920	0.81970	0.0570*	
H26A	0.79680	1.11660	0.71010	0.0960*	
H26B	0.86920	1.26040	0.72130	0.0960*	
H26C	0.75340	1.21980	0.76390	0.0960*	

### Atomic displacement parameters (Å^2^)

**Table:**

	*U*^11^	*U*^22^	*U*^33^	*U*^12^	*U*^13^	*U*^23^
Br1	0.0375 (3)	0.0761 (5)	0.0421 (3)	0.0165 (3)	0.0082 (2)	−0.0099 (3)
N1	0.023 (2)	0.035 (3)	0.0199 (19)	0.0012 (18)	0.0009 (16)	0.0006 (17)
N2	0.029 (2)	0.036 (3)	0.0225 (19)	0.0029 (19)	0.0050 (17)	−0.0002 (18)
C1	0.024 (2)	0.032 (3)	0.024 (2)	−0.001 (2)	0.0039 (19)	0.002 (2)
C2	0.030 (2)	0.027 (3)	0.023 (2)	0.002 (2)	0.004 (2)	0.004 (2)
C3	0.027 (2)	0.035 (3)	0.025 (2)	0.001 (2)	0.007 (2)	0.002 (2)
C4	0.027 (2)	0.040 (3)	0.019 (2)	0.000 (2)	0.0001 (19)	−0.002 (2)
C5	0.035 (3)	0.042 (4)	0.022 (2)	0.007 (2)	0.001 (2)	0.000 (2)
C6	0.036 (3)	0.035 (4)	0.028 (2)	0.007 (2)	−0.003 (2)	−0.001 (2)
C7	0.027 (2)	0.044 (4)	0.031 (3)	0.000 (2)	0.002 (2)	−0.014 (2)
C8	0.030 (3)	0.055 (4)	0.022 (2)	0.001 (3)	0.006 (2)	0.001 (2)
C9	0.034 (3)	0.030 (3)	0.023 (2)	0.006 (2)	0.003 (2)	−0.003 (2)
C10	0.028 (2)	0.037 (4)	0.021 (2)	−0.003 (2)	0.0027 (19)	0.000 (2)
C11	0.041 (3)	0.045 (4)	0.035 (3)	0.006 (3)	0.000 (2)	−0.003 (2)
C12	0.046 (3)	0.076 (5)	0.038 (3)	0.012 (3)	−0.011 (2)	−0.001 (3)
C13	0.032 (3)	0.077 (5)	0.051 (3)	0.011 (3)	−0.001 (3)	0.017 (3)
C14	0.042 (3)	0.053 (4)	0.044 (3)	0.012 (3)	0.008 (2)	0.008 (3)
C15	0.032 (3)	0.046 (4)	0.025 (2)	0.004 (2)	−0.002 (2)	0.003 (2)
C16	0.031 (3)	0.034 (3)	0.021 (2)	0.007 (2)	0.0010 (19)	0.003 (2)
C17	0.035 (3)	0.058 (4)	0.035 (3)	0.000 (3)	0.005 (2)	−0.009 (3)
C18	0.044 (3)	0.077 (5)	0.038 (3)	0.006 (3)	0.015 (3)	−0.008 (3)
C19	0.061 (4)	0.061 (5)	0.023 (3)	0.018 (3)	0.011 (3)	−0.002 (3)
C20	0.069 (4)	0.043 (4)	0.028 (3)	0.001 (3)	−0.002 (3)	−0.009 (2)
C21	0.054 (3)	0.031 (3)	0.025 (2)	0.001 (3)	0.006 (2)	0.004 (2)
C22	0.023 (2)	0.042 (4)	0.026 (2)	0.003 (2)	−0.0008 (19)	0.000 (2)
C23	0.032 (2)	0.038 (3)	0.024 (2)	−0.003 (2)	0.0048 (19)	0.001 (2)
C24	0.036 (3)	0.045 (4)	0.027 (2)	0.004 (3)	0.002 (2)	0.004 (2)
C25	0.043 (3)	0.064 (5)	0.037 (3)	0.003 (3)	0.005 (2)	0.020 (3)
C26	0.071 (4)	0.069 (5)	0.052 (3)	0.007 (4)	0.008 (3)	0.023 (3)

### Geometric parameters (Å, º)

**Table:**

Br1—C7	1.893 (4)	C23—C24	1.509 (6)
N1—C1	1.368 (5)	C24—C25	1.497 (7)
N1—C3	1.365 (6)	C25—C26	1.516 (7)
N1—C22	1.478 (5)	C5—H5	0.9500
N2—C1	1.313 (5)	C6—H6	0.9500
N2—C2	1.372 (5)	C8—H8	0.9500
C1—C4	1.454 (6)	C9—H9	0.9500
C2—C3	1.376 (6)	C11—H11	0.9500
C2—C10	1.443 (6)	C12—H12	0.9500
C3—C16	1.477 (6)	C13—H13	0.9500
C4—C5	1.399 (6)	C14—H14	0.9500
C4—C9	1.382 (6)	C15—H15	0.9500
C5—C6	1.365 (6)	C17—H17	0.9500
C6—C7	1.364 (6)	C18—H18	0.9500
C7—C8	1.372 (7)	C19—H19	0.9500
C8—C9	1.380 (6)	C20—H20	0.9500
C10—C11	1.392 (6)	C21—H21	0.9500
C10—C15	1.374 (7)	C22—H22A	0.9900
C11—C12	1.378 (7)	C22—H22B	0.9900
C12—C13	1.363 (8)	C23—H23A	0.9900
C13—C14	1.386 (7)	C23—H23B	0.9900
C14—C15	1.383 (6)	C24—H24A	0.9900
C16—C17	1.384 (6)	C24—H24B	0.9900
C16—C21	1.369 (7)	C25—H25A	0.9900
C17—C18	1.385 (7)	C25—H25B	0.9900
C18—C19	1.355 (8)	C26—H26A	0.9800
C19—C20	1.375 (7)	C26—H26B	0.9800
C20—C21	1.393 (6)	C26—H26C	0.9800
C22—C23	1.486 (6)		
			
C1—N1—C3	107.4 (3)	C4—C9—H9	120.00
C1—N1—C22	126.8 (4)	C8—C9—H9	120.00
C3—N1—C22	125.2 (3)	C10—C11—H11	120.00
C1—N2—C2	106.8 (3)	C12—C11—H11	120.00
N1—C1—N2	110.6 (4)	C11—C12—H12	120.00
N1—C1—C4	125.9 (4)	C13—C12—H12	120.00
N2—C1—C4	123.1 (4)	C12—C13—H13	120.00
N2—C2—C3	109.1 (4)	C14—C13—H13	120.00
N2—C2—C10	120.7 (3)	C13—C14—H14	120.00
C3—C2—C10	130.1 (4)	C15—C14—H14	120.00
N1—C3—C2	106.1 (4)	C10—C15—H15	119.00
N1—C3—C16	122.0 (4)	C14—C15—H15	119.00
C2—C3—C16	131.9 (4)	C16—C17—H17	120.00
C1—C4—C5	124.2 (4)	C18—C17—H17	120.00
C1—C4—C9	117.7 (4)	C17—C18—H18	120.00
C5—C4—C9	118.1 (4)	C19—C18—H18	120.00
C4—C5—C6	121.2 (4)	C18—C19—H19	120.00
C5—C6—C7	119.2 (4)	C20—C19—H19	120.00
Br1—C7—C6	119.7 (3)	C19—C20—H20	120.00
Br1—C7—C8	118.7 (3)	C21—C20—H20	120.00
C6—C7—C8	121.6 (4)	C16—C21—H21	120.00
C7—C8—C9	119.1 (4)	C20—C21—H21	120.00
C4—C9—C8	120.8 (4)	N1—C22—H22A	109.00
C2—C10—C11	119.7 (4)	N1—C22—H22B	109.00
C2—C10—C15	122.2 (4)	C23—C22—H22A	109.00
C11—C10—C15	118.0 (4)	C23—C22—H22B	109.00
C10—C11—C12	120.6 (5)	H22A—C22—H22B	108.00
C11—C12—C13	120.9 (5)	C22—C23—H23A	109.00
C12—C13—C14	119.4 (5)	C22—C23—H23B	109.00
C13—C14—C15	119.7 (5)	C24—C23—H23A	109.00
C10—C15—C14	121.4 (4)	C24—C23—H23B	109.00
C3—C16—C17	119.2 (4)	H23A—C23—H23B	108.00
C3—C16—C21	122.1 (4)	C23—C24—H24A	109.00
C17—C16—C21	118.8 (4)	C23—C24—H24B	109.00
C16—C17—C18	120.2 (5)	C25—C24—H24A	109.00
C17—C18—C19	120.6 (5)	C25—C24—H24B	109.00
C18—C19—C20	120.2 (4)	H24A—C24—H24B	108.00
C19—C20—C21	119.4 (5)	C24—C25—H25A	109.00
C16—C21—C20	120.9 (5)	C24—C25—H25B	109.00
N1—C22—C23	111.3 (3)	C26—C25—H25A	109.00
C22—C23—C24	112.4 (4)	C26—C25—H25B	109.00
C23—C24—C25	113.5 (4)	H25A—C25—H25B	108.00
C24—C25—C26	113.1 (4)	C25—C26—H26A	109.00
C4—C5—H5	119.00	C25—C26—H26B	110.00
C6—C5—H5	119.00	C25—C26—H26C	110.00
C5—C6—H6	120.00	H26A—C26—H26B	109.00
C7—C6—H6	120.00	H26A—C26—H26C	109.00
C7—C8—H8	120.00	H26B—C26—H26C	109.00
C9—C8—H8	120.00		
			
C3—N1—C1—N2	−1.2 (5)	C1—C4—C5—C6	179.0 (4)
C3—N1—C1—C4	−173.4 (4)	C9—C4—C5—C6	−2.3 (7)
C22—N1—C1—N2	−173.0 (4)	C1—C4—C9—C8	−179.5 (4)
C22—N1—C1—C4	14.9 (7)	C5—C4—C9—C8	1.6 (7)
C1—N1—C3—C2	0.8 (5)	C4—C5—C6—C7	0.8 (7)
C1—N1—C3—C16	−178.5 (4)	C5—C6—C7—Br1	178.7 (3)
C22—N1—C3—C2	172.8 (4)	C5—C6—C7—C8	1.2 (7)
C22—N1—C3—C16	−6.5 (7)	Br1—C7—C8—C9	−179.3 (3)
C1—N1—C22—C23	93.6 (5)	C6—C7—C8—C9	−1.8 (7)
C3—N1—C22—C23	−76.8 (5)	C7—C8—C9—C4	0.3 (7)
C2—N2—C1—N1	1.0 (5)	C2—C10—C11—C12	178.8 (4)
C2—N2—C1—C4	173.5 (4)	C15—C10—C11—C12	0.1 (7)
C1—N2—C2—C3	−0.5 (5)	C2—C10—C15—C14	−178.3 (4)
C1—N2—C2—C10	−179.9 (4)	C11—C10—C15—C14	0.3 (7)
N1—C1—C4—C5	−47.0 (7)	C10—C11—C12—C13	0.1 (8)
N1—C1—C4—C9	134.3 (5)	C11—C12—C13—C14	−0.6 (8)
N2—C1—C4—C5	141.8 (5)	C12—C13—C14—C15	1.0 (7)
N2—C1—C4—C9	−36.9 (7)	C13—C14—C15—C10	−0.9 (7)
N2—C2—C3—N1	−0.2 (5)	C3—C16—C17—C18	179.5 (5)
N2—C2—C3—C16	179.0 (5)	C21—C16—C17—C18	−1.0 (7)
C10—C2—C3—N1	179.1 (5)	C3—C16—C21—C20	179.9 (4)
C10—C2—C3—C16	−1.7 (9)	C17—C16—C21—C20	0.4 (7)
N2—C2—C10—C11	−29.8 (6)	C16—C17—C18—C19	1.6 (8)
N2—C2—C10—C15	148.8 (4)	C17—C18—C19—C20	−1.6 (8)
C3—C2—C10—C11	150.9 (5)	C18—C19—C20—C21	0.9 (8)
C3—C2—C10—C15	−30.5 (8)	C19—C20—C21—C16	−0.3 (8)
N1—C3—C16—C17	115.2 (5)	N1—C22—C23—C24	−169.5 (3)
N1—C3—C16—C21	−64.3 (6)	C22—C23—C24—C25	178.1 (4)
C2—C3—C16—C17	−63.9 (7)	C23—C24—C25—C26	−176.0 (4)
C2—C3—C16—C21	116.6 (6)		

## References

[bb1] Akkurt, M., Mohamed, S. K., Singh, K., Marzouk, A. A. & Abdelhamid, A. A. (2013). *Acta Cryst.* E**69**, o846–o847. 10.1107/S1600536813011446 PMC3684931 23795033

[bb2] Bruker (2011). *APEX2*, *SAINT* and *SADABS* Bruker AXS Inc., Madison, Wisconsin, USA.

[bb3] Farrugia, L. J. (2012). *J. Appl. Cryst.* **45**, 849–854.

[bb4] Hermann, W. A. & Kocher, C. (1997). *Angew. Chem. Int. Ed. Engl.* **36**, 2162–2187.

[bb5] Maier, T., Schmierer, R., Bauer, K., Bieringer, H., Buerstell, H. & Sachse, B. (1989*a*). US Patent 4820335.

[bb6] Maier, T., Schmierer, R., Bauer, K., Bieringer, H., Buerstell, H. & Sachse, B. (1989*b*). *Chem. Abstr.* **111**, 19494.

[bb7] Mohamed, S. K., Akkurt, M., Singh, K., Marzouk, A. A. & Abdelhamid, A. A. (2013). *Acta Cryst.* E**69**, o1243. 10.1107/S1600536813018229 PMC3793746 24109333

[bb8] Sheldrick, G. M. (2008). *Acta Cryst.* A**64**, 112–122. 10.1107/S0108767307043930 18156677

[bb9] Simpson, J., Mohamed, S. K., Marzouk, A. A., Talybov, A. H. & Abdelhamid, A. A. (2013). *Acta Cryst.* E**69**, o5–o6. 10.1107/S1600536812049100 PMC3588259 23476433

[bb10] Spek, A. L. (2009). *Acta Cryst.* D**65**, 148–155. 10.1107/S090744490804362X PMC2631630 19171970

[bb11] Welton, T. (1999). *Chem. Rev.* **99**, 2071–2084. 10.1021/cr980032t 11849019

